# Wide-Angle Mini-Light-Emitting Diodes without Optical Lens for an Ultrathin Flexible Light Source

**DOI:** 10.3390/mi13081326

**Published:** 2022-08-16

**Authors:** Yen-Lung Chen, Wen-Chung Chin, Chun-Wei Tsai, Chang-Che Chiu, Ching-Ho Tien, Zhi-Ting Ye, Pin Han

**Affiliations:** 1 Graduate Institute of Precision Engineering, National Chung Hsing University, Taichung 402, Taiwan; 2 Department of R&D Center, TOP RAYS Co., Ltd., Taichung 402, Taiwan; 3 Department of R&D Center, i-Wavefront Technology Ltd., New Taipei City 231625, Taiwan; 4 Department of Mechanical Engineering, Advanced Institute of Manufacturing with High-Tech Innovations, National Chung Cheng University, Taichung 402, Taiwan; 5 Department of Electronic Engineering, Lunghwa University of Science and Technology, Taoyuan 333326, Taiwan

**Keywords:** wide angle, mini-light-emitting diodes, flexible, luminous intensity curve, QLED display

## Abstract

This report outlines a proposed method of packaging wide-angle (WA) mini-light-emitting diode (mini-LED) devices without optical lenses to create a highly efficient, ultrathin, flexible planar backlight for portable quantum dot light-emitting diode (QLED) displays. Since the luminous intensity curve for mini-LEDs generally recommends a beam angle of 120°, numerous LEDs are necessary to achieve a uniform surface light source for a QLED backlight. The light-guide layer and diffusion layer were packaged together on a chip surface to create WA mini-LEDs with a viewing angle of 180°. These chips were then combined with a quantum dot (QD) film and an optical film to create a high-efficiency, ultrathin, flexible planar light source with excellent color purity that can be used as a QLED display backlight. A 6 in (14.4 cm) light source was used as an experimental sample. When 1.44 W was supplied to the sample, the 3200-piece WA mini-LED with a flexible planar QLED display had a beam angle of 180° on the luminous intensity curve, a planar backlight thickness of 0.98 mm, a luminance of 10,322 nits, and a luminance uniformity of 92%.

## 1. Introduction

Over the past decade, scientists have made substantial efforts to develop highly efficient, energy-saving, white-light-emitting diodes (LEDs) with high color saturation and long lifetimes, for flexible lighting and display technologies [1,2,3]. Colloidal quantum dots (QDs) are competitive candidates for next-generation illumination technologies and could replace current liquid crystal displays (LCDs) and organic LED (OLED) displays due to their superior photoluminescence, narrow-spectrum emission, high quantum efficiency, flexibility, and color-tuning capabilities. These advanced qualities in QDs could allow for smaller pixel sizes in micro-LED displays [4,5,6,7,8]. White-light-emitting materials are widely used in lighting and planar illuminator applications [9,10,11], and typically utilize inorganic phosphors containing rare earth elements (REEs) as their color conversion material. The current solution is not sustainable, as mining and utilization of REEs cause environmental and economic problems [12,13].

The development of REE-free color conversion materials with high efficiency, stability, and flexibility is a common research topic. In recent years, thin, flexible, and self-emissive OLED materials have been used for flexible lighting and displays, but their reliability is problematic and they have issues related to low driving currents [14,15]. Current research has improved QD technologies by improving their efficiency, reliability, and tunability with respect to emission colors [16,17,18,19]. Portable consumer electronics require lighting elements to be lightweight, thin, and flexible, so researchers have proposed designs using blue or ultraviolet chips with colloidal QD films as flexible planar light-source modules [6,7,20,21,22,23,24]. Research on high-color-gamut displays indicates that hybrid-type LEDs based on perovskite QDs (PQDs) may be a viable solution for wide-color-gamut display backlights. Mixed PQDs containing high-stability mesoporous silica nanocomposites with a wide color gamut, as well as white LEDs with QD color converters, have been used as backlights for display applications [25,26,27]. In mini-LED displays, drive-current algorithms have been applied to maximize the color gamut and high dynamic range [28,29,30].

Micro-LED displays have been found to have innate performance advantages over LCD displays, including higher contrast ratios, lower latency, advanced color saturation, intrinsic self-illumination, and higher efficiency. Despite their advantages, there are several factors preventing them from being mass produced, including inspection requirements, poor mass transfer yield, chip leakage currents, angular color shifts, and highly difficult repairs [31,32,33,34,35,36]. Conversely, QLED displays can provide the same perfect black as OLEDs, have high color saturation without burn-in, and have higher brightness than LCD displays [7,37,38,39]. QLED displays developed in relevant research have used conventional side-lit backlights, but have drawbacks such as low brightness, thick modules, inflexibility, and a lack of local dimming capabilities that prevent their use in practical applications [40,41,42,43]. Zhi Ting Ye et al. have previously developed a large-angle, blue-chip, scale package LED design but have not explored solutions that combine quantum dot films for mobile phone backlight modules [44]. Using different chip sizes will lead to different optimized package structure sizes. Therefore, the light distribution will also be different.

Herein, we propose a method of packaging WA mini-LEDs with a quantum dot (QD) film to create an ultrathin, flexible, planar light source that can be used as a backlight for portable QLED displays, while greatly reducing the number of LEDs required for the same area compared to the previous packaging requirements.

## 2. Fabrication of Packaged Wide-Angle Mini-LEDs

**Figure 1:**
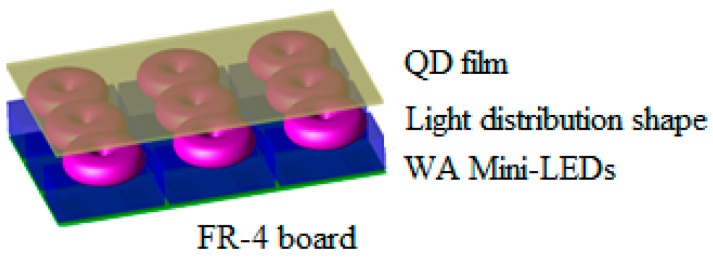
Three-dimensional structure of WA mini-LEDs used on an ultrathin, flexible, planar light source as a backlight for portable QLED displays.

WA mini-LED chips were based on GaN flip-chip blue LEDs with an emission wavelength of 450 nm. The length, width, and height of these chips were 228.6, 127, and 150 μm, respectively. The GaN flip-chip blue LEDs were obtained from Harvatek Corporation, Hsinchu City, Taiwan. Mini-chips are advantageous because they do not need wire bonding, they are capable of withstanding high current densities, there is no lead frame requirement, and their packages have decreased thermal resistance. Figure 1 shows the 3D structure of the WA mini-LED chips used on an ultrathin, flexible, planar light source as a backlight for portable QLED displays.

**Figure 2:**
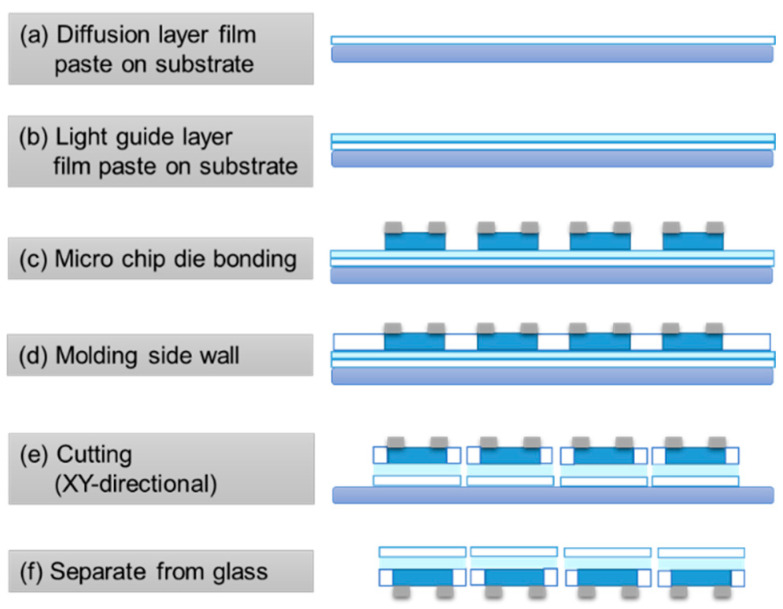
Process flow for packaged wide-angle mini-LEDs.

Figure 2 presents the WA square mini-LED chips’ scale packaging process [44]. The first step in the packaging process involved depositing a diffusion layer on a glass substrate. The source material’s composition was 5% titanium dioxide (TiO_2_) particles and 95% silicone resin composite glue, with a refractive index of ~1.56. The silicone resin used was Dow Corning OE-7662, and the refractive index of silicone resin is 1.55.

The diffusion layer was used to control the transmission–reflection ratio. The second step involved depositing a light-guide layer on top of the diffusion layer, with the silicone resin functioning as the source material [44]. The light-guide layer was used to control the light extraction efficiency and light distribution shape. The third step was to arrange the microscale chips and perform die bonding. Great care must be taken to preserve the appropriate spacing between the microscale chips. The fourth step was to apply the TiO_2_/silicone resin composite glue to mold a side wall around the microscale chips and then cut the WA mini-LEDs. The added side wall was used to prevent transverse blue-light emission. The final step involved separating the WA mini-LEDs from the glass substrate through ultraviolet (UV) exposure. The resulting WA mini-LED square package dimensions were 800 × 800 × 580 µm^3^. Not only does this process substantially reduce the number of light sources used, it also produces light sources that are highly efficient, flexible, and thin. These findings imply that the WA mini-LED square packages produced using these methods are suitable backlights for advanced QLED display applications.

## 3. Fabrication of QD Film

The TiO_2_ particles and PET films were provided by Harvatek Corporation. A QD film (6 in (14.4 cm)) was prepared by dispersing commercial green QDs (λg ≈ 530 nm) and red QDs (λr ≈ 626 nm) in a UV-cured poly (methyl methacrylate) (PMMA) polymer. The UV-cured poly (methyl methacrylate) transparent adhesive used was Vitralit^®^ 1655.

**Figure 3:**
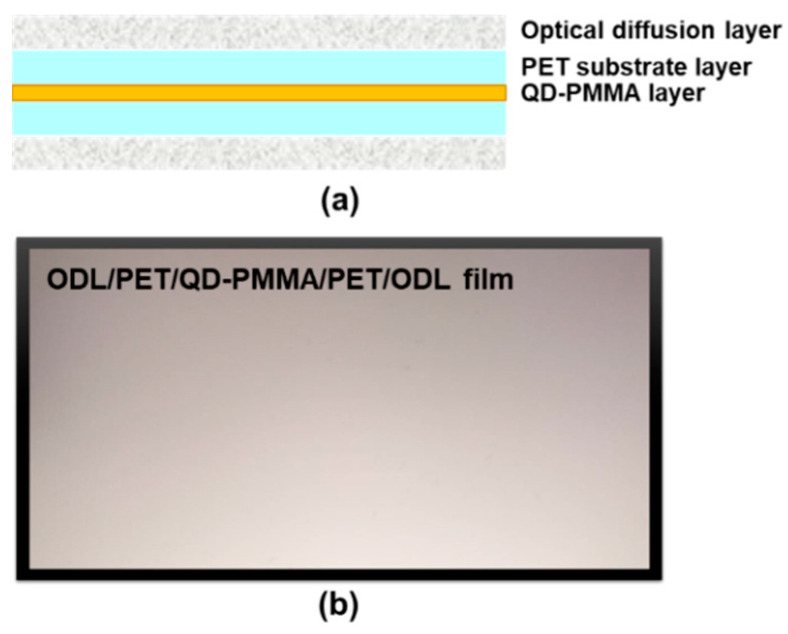
(**a**) Structural diagram and (**b**) photograph of the QD film.

The CdSe-based QDs were from Unique Materials Co., Ltd. and had weight ratios of about 20:1 (polymer: QD). The hybrid QD–PMMA monomer layers were coated on two poly (ethylene terephthalate) (PET) films as covering layers, using an automatic blade coater to produce sandwich-like structures. The PET/QD–PMMA/PET film was then laminated and cured through irradiation with 365 nm UV light for 30 s. Finally, a doctor-blade coater was used to coat the optical diffusion layer (ODL) onto the PET/QD–PMMA/PET film to form an ODL/PET/QD–PMMA/PET/ODL film [45], as shown in Figure 3.

## 4. Fabrication of Flexible Circuit Board Design for Wide-Angle Mini-LEDs

**Figure 4:**
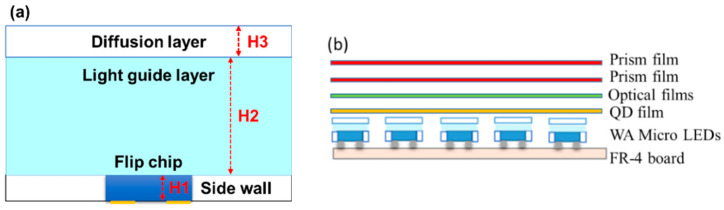
Schematic of the 2D mini-LED backlight-unit structure: (**a**) 2D WA mini-LED structure; (**b**) WA mini-LED backlight-unit structure.

An epoxy glass fiber unclad laminate (FR4) board was used as a flexible circuit board, and its length, width, and thickness were 131.2, 67.2 and 0.4 mm, respectively. The active area of the sample was 127.2 × 63.2 mm^2^, and the pitch was 0.80 mm. A total of 3200 packaged WA mini-LEDs were mounted in an array onto a flexible FR4 board, as shown in Figure 4a. The sidewall of the resin is wrapped around the chip, and the reflectivity of the resin is 92%. Figure 4b details the structure of the backlight unit consisting of 80 × 40 arrays of square-shaped packaged WA mini-LEDs, with a QD film, optical film, and prism film. The optical film and prism film used 3M TBEF2 and UDF2-35, respectively. All of these features combined produce an ultrathin, flat, and uniform light source. Each layer of optical film was stacked in sequence, with minimal air spacing between each layer [44].

## 5. Results and Discussion

Analysis of the ultra-thin, flat, uniform WA mini-LED light source gave the following results. The light-guide layer was well correlated with the light extraction efficiency. The diffusion layer was used to control the ratio of the light emittance rate to the reflected light rate. The mini-LED central light intensity *I_N_* is defined in Equation (1).

(1) $$I_{N}=\frac{Central\;intersity\;value\;of\;full\;angle\;\left(I_{C}\right)}{peak\;intensity\;value\;of\;full\;angle\;\left(I_{p}\right)}\times 100\%\;$$

**Figure 5:**
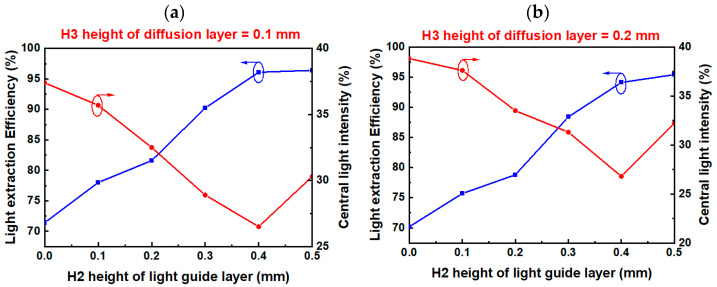
Schematic of the optical mode structure and the light extraction efficiency and center intensity for different light-guide layer (H2) heights, at diffuser layer (H3) heights of (**a**) 0.1 mm and (**b**) 0.2 mm for the packaged WA mini-LED.

The height of the diffusion layer (H3) was initially fixed at 0.1 mm. Without a light-guide layer, the light extraction efficiency and central light intensity were 71.4% and 37.4%, respectively. When the height of the light-guide layer (H2) was increased from 0.1 to 0.4 mm, the light extraction efficiency was observed to increase from 78% to 96.1%, whereas the central light intensity decreased from 35.7% to 26.5%, as shown in Figure 5a.

When the height of the diffusion layer (H3, with no light-guide layer) was fixed at 0.2 mm, the light extraction efficiency decreased to 70.2% and the central light intensity increased to 38.8%. Similarly, when the height of the light-guide layer (H2) was increased from 0.1 to 0.4 mm, the light extraction efficiency increased from 75.7% to 94.1%, whereas the central light intensity decreased from 37.6% to 26.8, as shown in Figure 5b.

Based on these results, the optimal values for the height of the light-guide layer (H2) and the height of the diffusion layer (H3) were determined to be 0.4 and 0.1 mm, respectively. These parameters were subsequently applied to the light source of the flexible planar QLED display.

**Figure 6:**
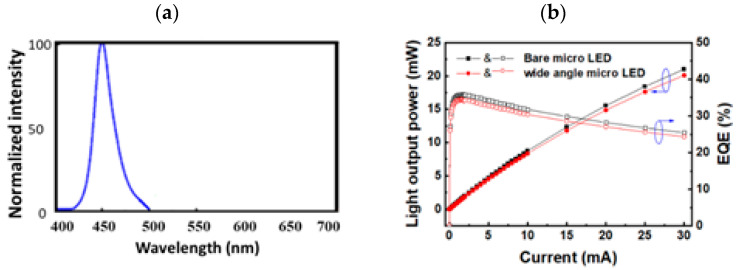
(**a**) The spectrum of the WA mini-LED and (**b**) the L–I curves of the bare mini-LED and packaged WA mini-LED.

Figure 6a presents the spectrum of the WA mini-LED’s wavelength versus the normalized intensity. Figure 6b presents the measured light-current (L-I) and external quantum efficiency (EQE) characteristics for a bare mini-LED and the packaged WA mini-LED. The light output power of the two LEDs was only slightly different, with the packaged WA mini-LED experiencing a 4.20% deterioration in light output power compared to the bare mini-LED at an injection current of 15 mA. The light loss of the WA mini-LED was attributed to the addition of a diffusion layer on top of the light-guide layer, causing some light to be absorbed.

**Figure 7:**
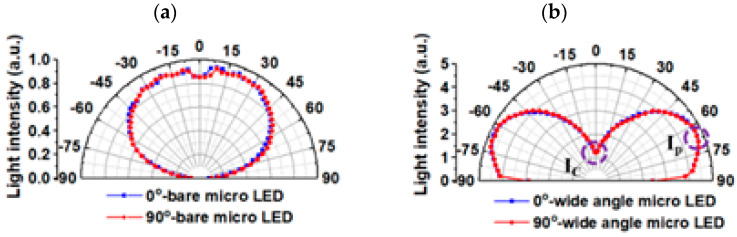
The 2D light distribution patterns of (**a**) the bare mini-LED and (**b**) the packaged WA mini-LED.

Figure 7 display the 2D light distribution patterns of the bare mini-LED and the packaged WA mini-LED at an injection current of 10 mA. The bare mini-LED and packaged WA mini-LED viewing angles were measured to be 147° and 180°, respectively.

Some light in the device was diffusely reflected by the diffusion layer, escaping from the side wall of the light-guide layer, so the center intensity (*I_C_*) of the WA mini-LED was reduced to 26.5% and the peak angle (*I_p_*) of the WA mini-LED was enhanced to 67°. Therefore, the hot-spot phenomenon directly above the light source can be reduced, improving the uniformity.

**Figure 8:**
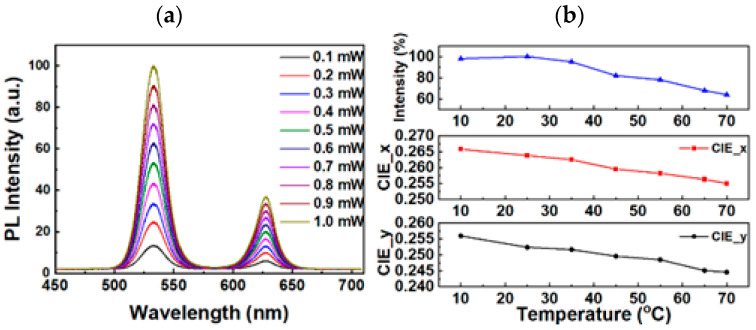
(**a**) PL intensity as a function of laser excitation power for QD films. (**b**) Relative PL intensity values and the two CIE color coordinates x and y for different QD film temperatures.

The photoluminescence (PL) intensities of QD films were tested at different laser excitation powers, using a 365 nm laser. Figure 8a displays the laser-excitation-power-dependent PL spectra of the QD films. The positions of their peak wavelengths at full width at half maximum (FWHM) were almost the same across the range of excitation powers from 0.1–1.0 mW, indicating that these peaks were relatively stable under different laser excitation powers. Two excitation bands were observed with peaks at 532 and 626 nm. The FWHM values corresponding to these peaks were 24.4 and 23.2 nm, respectively. The two International Commission on Illumination (CIE) color coordinates x and y as functions of temperature were measured from 10 to 70 °C, with respect to the relative PL intensity values. These results are depicted in Figure 8b. As the temperature increased, the observed relative PL intensity value decreased, and a slight but distinguishable progressive blue shift of the two CIE color coordinates x and y was observed.

**Figure 9:**
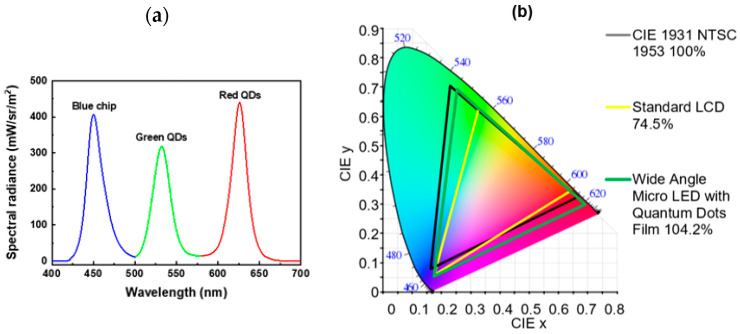
(**a**) EL spectra. (**b**) Color gamut in operation photograph of WA mini-LED flexible planar QLED backlight.

The electroluminescence (EL) spectra of the individual red–green–blue (RGB) colors for the WA mini-LED flexible planar QLED backlight are shown in Figure 9. The emission peak wavelengths were 626, 532, and 450 nm, and the FWHM values were 23.2, 24.4, and 24.8 nm, respectively. The display had good monochromaticity, high color purity, and high color saturation. The EL spectra demonstrate that the RGB color coordinates of the WA mini-LED flexible planar QLED backlight were (0.6947, 0.3028), (0.2347, 0.6991), and (0.1525, 0.0535) in the CIE 1931 chromaticity diagram. The display can thus realize a wider color gamut due to the narrow EL spectra, as shown in Figure 9b. The color-gamut coverage rate of the WA mini-LED flexible planar QLED backlight reached 104.2% of the NTSC 1931 color space, which is sufficient for full-color performance in displays.

**Figure 10:**
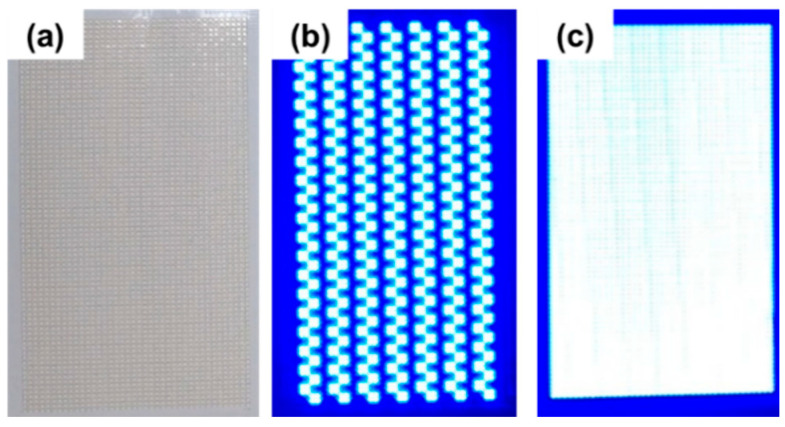
(**a**) WA mini-LEDs with flexible planar backlight module, (**b**) local dimming, and (**c**) all mini-LEDs activated.

Figure 10a presents the 3200-piece WA mini-LED flexible planar backlight module. A Yongtek Electronics die bonder was used to complete the process. Figure 10b presents an image from the local dimming function that was obtained by using a driver integrated circuit to control the mini-LEDs in specific areas. Figure 10c shows an image of the model when all the mini-LEDs were activated. This model had an operational voltage of 96.2 V, an operational current of 0.015 A, and a power consumption of 1.44 W.

**Figure 11:**
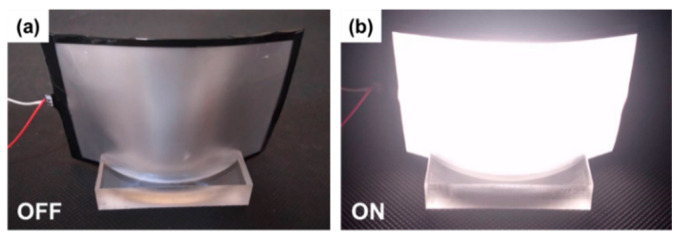
Photographs of the flexible WA mini-LED planar QLED display: (**a**) shut down and (**b**) in operation.

The images in Figure 11 are photographs of the WA mini-LED flexible planar QLED backlight. Image (**a**) shows the display in the off state, and image (**b**) shows the display in operation.

Table 1 displays the optoelectronic properties of the WA mini-LED flexible planar QLED backlight. These results demonstrate that the illuminance distribution was uniform when the display was bent. The display had a luminance of 10,322 nits and a brightness uniformity of 92%, while drawing only 1.44 W of power. A full-array emission backlight unit has some advantages when WA mini-LEDs are used. WA mini-LEDs are small and have larger emission angles and illumination areas, thus improving backlight uniformity and reducing the number of LEDs necessary to illuminate an area. These properties allow for the development of ultrathin, flexible, and low-power QLED displays.

**Figure 12:**
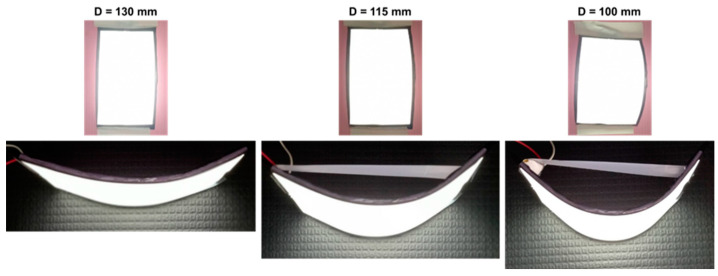
Emission photographs of the wide-angle micro-LED flexible planar QLED display at different bending diameters.

Figure 12 demonstrates that the wide-angle micro-LED flexible planar QLED display had satisfactory performance metrics when bent to different curvatures. The bending diameters of the display ranged from 130 to 100 mm. The brightness uniformity was not affected by the bending curvature.

Table 2 shows the luminance values of the display at different bending diameters. For bending diameters from 130 to 100 mm, the voltage had minimal variations when a 0.015 A current was applied. A decrease in brightness of only 2.57% was observed. Thus, the display is suitable for flexible display applications.

**Figure 13:**
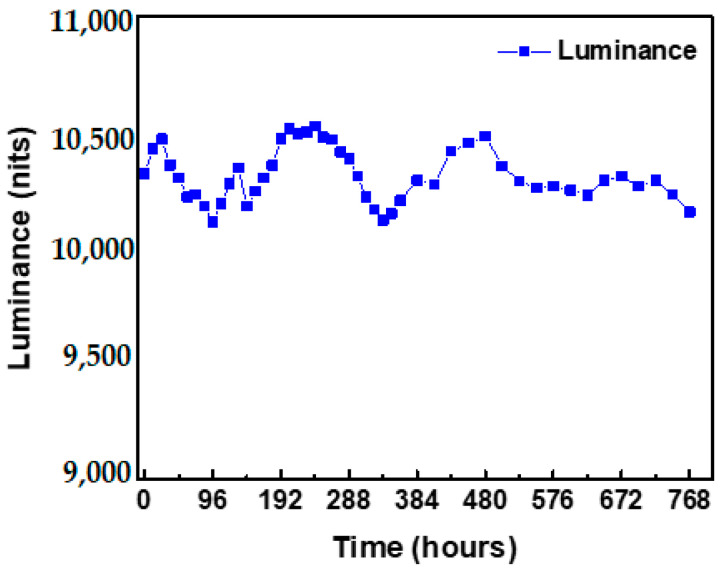
Burn-in test results of the WA mini-LED flexible planar QLED display.

Figure 13 displays the burn-in test results for the WA mini-LED flexible planar QLED backlight. During the 768 h test, the display was placed on a platform at 25 °C, and a 0.015 A current was applied. The resulting luminance values were consistently greater than 10,000 nits. The attenuation was 0.16% after 168 h and 1.6% after 768 h. These results indicate that the display is reliable.

## 6. Conclusions

This study analyzed the use of mini-LEDs for flexible mobile backlight displays. Using a novel packaging process, the performance of the wide-angle mini-LEDs was significantly improved. Compared with bare mini-LEDs, the light extraction efficiency reached 96.1% and the center intensity was reduced to 26.5%. This resulted in a larger emission angle, a larger illumination area, and a reduced quantity of LEDs required compared to unpackaged mini-LEDs with the same surface area. The wide-angle, mini-LED flexible planar QLED display had excellent optoelectronic properties, with a power consumption of 1.44 W, an ultrathin, flexible-light-source thickness of 0.98 mm, a luminance of 10322 nits, a brightness uniformity of 92%, and a wide color gamut reaching 104.2% of the NTSC 1931 standard for backlit displays. These results indicate that a wide color gamut can be achieved by applying this backlight-unit solution to future QLED displays.

## Figures and Tables

**Table 1:** Optoelectronic properties of the WA mini-LED flexible planar QLED backlight.

Item	Characteristics
Operational voltage (V)	96.2 V
Operational current (A)	0.015 A
Power consumption (W)	1.44 W
Luminance (nits)	10,322 nits
Uniformity (%)	92%
NTSC coverage (%)	104.2%

**Table 2:** Luminance values of the wide-angle micro-LED flexible planar QLED display at different bending diameters.

Bending Diameter (mm)	Operation Current (A)	Operation Voltage (V)	Luminance (nits)
Flat	0.015	96.2	10,322
130	0.015	96.4	10,128
115	0.015	96.3	10,056
100	0.015	96.3	10,097

## Data Availability

Data sharing is not available.
